# Successful reintervention using a novel steerable catheter after dislocation of a transluminal gallbladder stent

**DOI:** 10.1055/a-2436-6980

**Published:** 2024-10-25

**Authors:** Takeshi Ogura, Yuki Uba, Nobuhiro Hattori, Kimi Bessho, Hiroki Nishikawa

**Affiliations:** 1 Endoscopy Center, Osaka Medical and Pharmaceutical University Hospital, Takatsuki, Japan; 2 2nd Department of Internal Medicine, Osaka Medical and Pharmaceutical University, Takatsuki, Japan

Endoscopic ultrasound-guided gallbladder drainage (EUS-GBD) is indicated for acute cholecystitis in poor surgical candidates 1 2 . Although technical tips for EUS-GBD have been well established, long-term outcomes after stent removal remain unclear. If acute cholecystitis recurs after stent removal, reintervention through the fistula between the gallbladder and stomach or duodenum should be considered. To perform reintervention, the guidewire should be inserted into the gallbladder. However, in the transduodenal approach, this procedure might be challenging due to the limited space available to manipulate the echoendoscope. Recently, a novel steerable catheter (Zeon Medical, Tokyo, Japan) has become available in Japan 3 4 . This catheter can be manipulated upwards and downwards 90 degrees, and the fulcrum for the tip bend is closer to the tip (15 mm) ( Fig. 1 ). Therefore, guidewire access to a challenging site can be performed easily. Successful reintervention using the novel steerable catheter after EUS-GBD stent dislocation is described below.

**Fig. 1:**
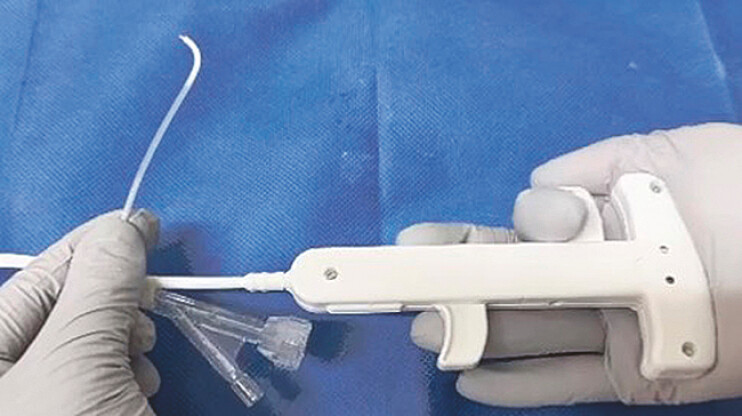
A novel steerable catheter (Zeon Medical, Tokyo, Japan).

An 88-year-old woman was admitted to our hospital with acute cholecystitis. She had undergone EUS-GBD from the duodenum for acute cholecystitis using a plastic stent 2 months earlier. However, computed tomography showed dislocation of the plastic stent. Therefore, reintervention was attempted.

A duodenoscope was inserted into the duodenum and the fistula was detected ( Fig. 2 ). Guidewire insertion into the gallbladder was attempted; however, because the angle between the fistula and the gallbladder was acute ( Fig. 3 **a** ), guidewire insertion failed. Guidewire insertion using the novel steerable catheter was then performed easily ( Fig. 3 **b** ). After guidewire deployment within the gallbladder, a double-lumen catheter was also inserted along this guidewire; then, an additional guidewire was deployed. Finally, two double-pigtail plastic stents were deployed without any adverse events ( Fig. 3 **c** , Video 1 ).

**Fig. 2:**
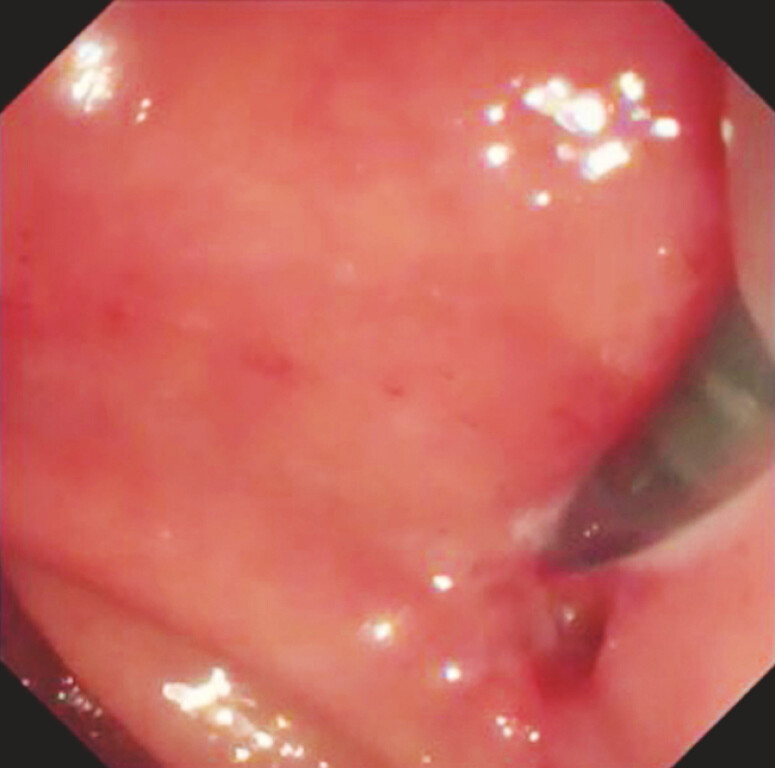
A duodenoscope was inserted into the duodenum, and a fistula was detected.

**Fig. 3:**
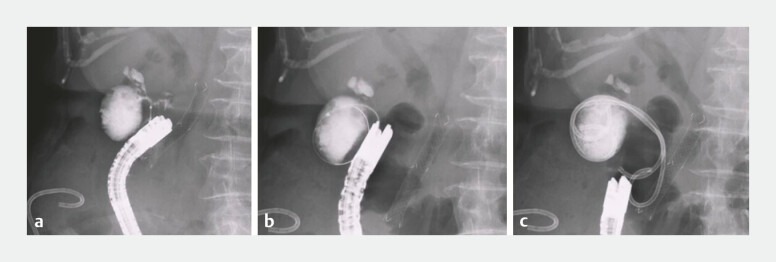
Guidewire insertion and stent placement. **a** Guidewire insertion into the gallbladder was attempted but failed due to the acute angle between the fistula and the gallbladder. **b** Guidewire insertion using the novel steerable catheter was then performed. **c** Two double-pigtail plastic stents were deployed without any adverse events.

Guidewire insertion using a novel steerable catheter was successfully performed.Video 1

In conclusion, this novel catheter could be a useful option for successful selective guidewire insertion.

Endoscopy_UCTN_Code_CPL_1AL_2AD

## Notes

Endoscopy E-Videos https://eref.thieme.de/e-videos
*E-Videos* is an open access online section of the journal *Endoscopy* , reporting on interesting cases and new techniques in gastroenterological endoscopy. All papers include a high-quality video and are published with a Creative Commons CC-BY license. Endoscopy E-Videos qualify for HINARI discounts and waivers and eligibility is automatically checked during the submission process. We grant 100% waivers to articles whose corresponding authors are based in Group A countries and 50% waivers to those who are based in Group B countries as classified by Research4Life (see: https://www.research4life.org/access/eligibility/ ). This section has its own submission website at https://mc.manuscriptcentral.com/e-videos .
